# Objectively measured patterns of sedentary time and physical activity in young adults of the Raine study cohort

**DOI:** 10.1186/s12966-016-0363-0

**Published:** 2016-03-24

**Authors:** Joanne A. McVeigh, Elisabeth A. H. Winkler, Erin K. Howie, Mark S. Tremblay, Anne Smith, Rebecca A. Abbott, Peter R. Eastwood, Genevieve N. Healy, Leon M. Straker

**Affiliations:** School of Physiotherapy and Exercise Science, Curtin University, Perth, WA Australia; The University of Queensland, School of Public Health, Brisbane, Australia; Children’s Hospital of Eastern Ontario Research Institute, Ottawa, Canada; ESMI, University of Exeter Medical School, St.Luke’s Campus, Exeter, UK; Centre for Sleep Science, School of Anatomy, Physiology & Human Biology, University of Western Australia, Perth, Western Australia Australia; Baker IDI Heart & Diabetes Institute Melbourne, Victoria, Australia

**Keywords:** Raine Study, Young adults, Physical activity, Patterns, Accumulation, Sedentary behaviour, Accelerometry

## Abstract

**Background:**

To provide a detailed description of young adults’ sedentary time and physical activity.

**Methods:**

384 young women and 389 young men aged 22.1 ± 0.6 years, all participants in the 22 year old follow-up of the Raine Study pregnancy cohort, wore Actigraph GT3X+ monitors on the hip for 24 h/day over a one-week period for at least one ‘valid’ day (≥10 h of waking wear time). Each minute epoch was classified as sedentary, light, moderate or vigorous intensity using 100 count and Freedson cut-points. Mixed models assessed hourly and daily variation; t-tests assessed gender differences.

**Results:**

The average (mean ± SD) waking wear time was 15.0 ± 1.6 h/day, of which 61.4 ± 10.1 % was spent sedentary, 34.6 ± 9.1 % in light-, 3.7 ± 5.3 % in moderate- and, 0.3 ± 0.6 % in vigorous-intensity activity. Average time spent in moderate to vigorous activity (MVPA) was 36.2 ± 27.5 min/day. Relative to men, women had higher sedentary time, but also higher vigorous activity time. The ‘usual’ bout duration of sedentary time was 11.8 ± 4.5 min in women and 11.7 ± 5.2 min in men. By contrast, other activities were accumulated in shorter bout durations. There was large variation by hour of the day and by day of the week in both sedentary time and MVPA. Evenings and Sundays through Wednesdays tended to be particularly sedentary and/or inactive.

**Conclusion:**

For these young adults, much of the waking day was spent sedentary and many participants were physically inactive (low levels of MVPA). We provide novel evidence on the time for which activities were performed and on the time periods when young adults were more sedentary and/or less active. With high sedentary time and low MVPA, young adults may be at risk for the life-course sequelae of these behaviours.

**Electronic supplementary material:**

The online version of this article (doi:10.1186/s12966-016-0363-0) contains supplementary material, which is available to authorized users.

## Background

Early adulthood (defined here as 20–25 years) is a critical period for establishing independence and adopting lifestyle behaviours important to physical and mental health, and is thus gaining recognition as an important time for health promotion and disease prevention efforts [1, 2]. Although some health-risk behaviours such as smoking and drinking tend to decrease with increased adult responsibility [2, 3], early adulthood has been identified as a period of increasing risk for reduced time spent in moderate- and vigorous-intensity physical activity (MVPA) [4, 5] and increased time spent in sedentary behaviours [6]. Both insufficient MVPA and excessive time spent sedentary have emerged as key risk factors for non-communicable disease [7, 8], and thus have important implications for long-term health. In Australia, insufficient MVPA (i.e. not meeting the physical activity guidelines of at least 30 min of MVPA on most days of the week), was estimated to be responsible for 6.6 % of the total burden of disease and injury in 2003 [9]. Similarly, excessive sedentary behaviour has been estimated to be responsible for 6.9 % of the mortality burden in Australia [10]. Thus, how sedentary and physically active young women and men are is important to their current and future health.

In addition to the total amount of time spent in sedentary behaviour and in physical activity (at light, moderate and vigorous intensities) being important for health outcomes in adults [11–13], the patterns of how physical activity and sedentary time are accumulated may also have implications for health. For example, frequent interruptions to sedentary time have been beneficially associated with indicators of adiposity in observational studies [14], and have resulted in acute benefits for glucose and insulin in laboratory trials [15]. Targeted interventions may thus benefit from a detailed understanding of when over the course of a day or week populations are most sedentary and least active.

The majority of studies on sedentary time and physical activity have been undertaken in children, middle-aged adults, and elderly adults, with few studies focussed on young adults. The available data over the period from late adolescence to early adulthood from either self-report measures [16–19] or objective measures [6, 20–22] suggests that (on average) there is a small decline in MVPA, and a substantial increase in sedentary time. A few studies with objective measures have included a proportion of young adults in samples across very broad age ranges [6, 22–24]. Of these studies, the closest to a young adult group examined has been from the NHANES study, which reported that young adults aged 20–29 years spend approximately 55 % of their waking day in sedentary time and 40 min per day in MVPA [6, 20]. However, these reports did not examine *how* sedentary time or physical activity were accumulated nor *when*, in terms of time of day or day of the week. Although these studies help to identify young adults as a potential key target group for prevention efforts, the pattern of how and when young adults accumulate sedentary behaviour and physical activity has not been well characterised. Thus, there is limited detailed information to help inform interventions targeting this group.

Therefore, the current study aimed to answer the following questions about young adults: (1) how sedentary and physically active are young men and women? and; (2) what are the patterns by which young adults accumulate sedentary time and physical activity, in terms of how much at a time, and when across the day and week they perform these behaviours?

## Methods

### Participants

The data for this study are from the 22 year-old follow-up of participants enrolled in the Western Australian Pregnancy Cohort (Raine) Study (www.rainestudy.org.au) [25]. Briefly, 2900 pregnant women attending a public antenatal clinic or nearby private practices in Perth, Australia were recruited into the study between May 1989 and November 1991. A total of 2868 children underwent serial assessments in utero, at birth and at ages 1, 2, 3, 5, 8, 10, 14, 17, 20 and 22 years. Of the 2868 participants originally enrolled, 2086 were still ‘active’ and contacted for participation in the 22 year follow up between 2012 and 2014. Of these, 1234 young adults took part in some aspect of the year 22 year follow up, with 926 participants agreeing to participate in the activity monitor component.

### Ethics

Informed, written consent to participate in the study was obtained by each participant. The study was approved by the institutional ethics committee of Curtin University (HR 23/2013) and the University of Western Australia (RA/4/1/2646 & RA/4/1/2100).

### Data collection

All participants attended a central testing site in Perth, Australia. Upon arrival, participants were asked to complete questionnaires and were then fitted with the hip-worn Actigraph GT3X+ (Actigraph, Pensacola, FL, USA) accelerometer and were asked to wear it continuously for the following eight days. Participants were also asked to wear another monitor on their non-dominant wrist: data from the wrist monitor will be presented elsewhere. Participants also completed several hours of clinical assessments and an overnight sleep study (for full protocol details see [26]).

### Demographic and anthropometric data

All participants completed a questionnaire composed of standard questions and scales drawn from prior studies to gather information on sex, ethnicity, income, living arrangements, education, working status, children, self-rated health and medical history [26]. Height and weight were taken with participants wearing indoor clothing without shoes and measured according to standardized methods by trained research assistants [26].

### Sedentary time and physical activity

Sedentary time and physical activity were objectively measured using a continuous wear (24 h/day) protocol over an approximately eight day period using the Actigraph GT3X+ accelerometer. The GT3X+ was programmed to record raw data at a frequency of 30Hz which were later reduced to vertical axis movement counts per 60 s epoch for the purpose of the current analyses. Participants were instructed to wear the accelerometer on the right hip continuously, except during bathing or aquatic activities. Participants were also provided with an activity/sleep diary and asked to record all sleep times, work or study times, and any accelerometer removal times over the assessment period. A reminder contact (email, SMS, or phone) was provided on day two of the wear period for each participant.

Accelerometer data were downloaded and processed in SAS (version 9.3, SAS Institute, Cary, NC, USA). Waking wear data could not be identified from diaries as only 41 % (380/926) of participants returned diaries with sufficient information. We trialled available low-burden methods for identifying waking wear in hip-worn minute epoch data (that have not been validated in young adults [27–31]), and an algorithm we developed relative to visual identification (two raters) in a subset of 95 participants. Our algorithm was used, as it was the most accurate available method for this sample [32, 33]. The agreement achieved was similar to the agreement between raters, with a mean difference between expert visual identification and the automated algorithm for waking wear time of 7 min/day (95 % Limits of Agreement: −220 to 234 min) on days deemed valid (defined as ≥10 h of waking wear time [34]) by both methods. Common cut-points [6, 35] were used to class each minute as sedentary (<100 counts per minute, cpm), light intensity (100–1951 cpm), moderate intensity (1952–5724 cpm), or vigorous intensity (>5724 cpm). Time spent at each activity intensity was calculated, as well as the average accelerometer counts at each activity intensity. As in previous studies [36, 37], we also measured MVPA in ≥10 min bouts, allowing for two minutes below the cut-point (modified 10 min bouts), and in strict bouts of MVPA ≥10 min (no hesitation allowed). Activities were summarised by bout duration, in hourly time blocks for each day, as totals for each day, and as averages across valid days. Based on the method proposed by Chastin and Granat [36], the ‘usual’ sedentary bout duration was also calculated for each individual. An individual accumulates half of all his or her sedentary time in bouts longer than his or her ‘usual’ bout duration. Usual bout durations were calculated from the midpoint of each individual’s sedentary accumulation curve, fitted by non-linear regression (Levenberg–Marquardt algorithm) as described by Chastin et al (Chastin et al 2015). The raw step count (uncensored, i.e. all steps counted) recorded per minute was also used.

### Statistical analyses

All participants with ≥1 valid day of data were included in the present analyses (*n* = 733). Statistical analyses were conducted using SAS (version 9.4, SAS Institute, Cary, NC, USA) with significance set at two-tailed *p* < 0.05. Amounts of sedentary time and physical activity, and usual sedentary bout duration were reported using descriptive statistics (e.g., means, medians, percentages) for the group overall, and for men and women separately. Comparisons between men and women were conducted using student’s t-test or Wilcoxon-Mann-Whitney test as appropriate.

For light activity and MVPA, which are more sporadic than sedentary time, the observed accumulation across bouts of increasingly long duration across the entire sample of young men and young women are reported. From these accumulation plots, the bout durations explaining approximately half the sample of young women’s and men’s time spent in these activities are reported. For comparison purposes, these are also reported for sedentary time. Accumulation of physical activity across categories of intensity and bout duration was also described using Exposure Variation Analysis (EVA) [38, 39].

Variation by hour of the day and by day of the week in MVPA, and the sedentariness of the remaining time not spent in MVPA (as sedentary/light ratio), were examined using linear mixed models (continuous outcomes) or generalised linear mixed models (binary outcomes). These models included all valid observed data and were adjusted for several sociodemographic characteristics to account for compositional differences in the groups providing data on certain days or hours. Hourly and daily sedentary/light ratio and daily MVPA data were log transformed. As many hours of data contained no MVPA, hourly MVPA was examined as ‘any MVPA’ yes/no. Variance-covariance structures were selected based on Bayes Information Criterion; unstructured variance-covariance was used for the daily models, and a Toepliz structure was used for the hourly models. From these models, differences between the day (or hour) and the grand mean are reported, with adjustment for multiple comparisons (Sidak method), based on least squares means estimations. Random day-to-day variability is also reported.

## Results

Of the 926 participants who agreed to participate in accelerometer data collection, 773 participants, 389 (50.3 %) women (22.1 ± 0.66 years) and 384 (49.7 %) men (22.1 ± 0.60 years) provided at least one day of valid accelerometer data with the overall median being six days of valid wear; 563 participants (73 %) provided ≥4 valid days. The physical, socio-demographic, and health-related characteristics of these participants are shown in Table 1. The 773 participants participating in the 22 year old Raine follow-up who had ≥ 1 valid day of accelerometer data, when compared with 2011 census data for similarly aged young adults in Western Australia, reflected usual study participation biases. There was an over-representation (≥10 %) of higher socioeconomic status in terms of education (more high school completion and current study), occupation (more professional women), and employment (more currently employed, though typically for fewer hours, consistent with more currently studying) (Additional file 1: Table S1).

**Table 1:** Characteristics of study participants

Variable	All (*n* = 773)	Women (*n* = 389)	Men (*n* = 384)
Physical			
Age (y)	22.1 ± 0.6	22.1 ± 0.7	22.1 ± 0.6
Weight (kg)	74.6 ± 16.9	69.1 ± 16.7	80.3 ± 15.2
Height (cm)	172.5 ± 9.6	166.0 ± 6.2	179.1 ± 7.7
Body Mass Index (kg/m^2^)	25.0 ± 5.1	25.1 ± 5.8	25.0 ± 4.3
Caucasian	651 (84.2)	328 (84.3)	323 (84.1)
Socio-demographic			
*Living arrangements*			
Live alone	16 (2.3)	10 (2.8)	6 (1.7)
Live with partner	111 (15.8)	40 (11.4)	45 (12.9)
Live with parents (not partner)	419 (59.8)	218 (62.1)	201 (57.4)
Live with others (not partner, not parents)	155 (22.1)	83 (23.7)	98 (28.0)
Has (or expecting) children	30 (5.0)	15 (6.3)	15 (6.0)
*Educational attainment*			
High school or less (not currently studying)	140 (20.1)	66 (19.0)	74 (21.2)
Currently studying, no qualification yet	349 (50.1)	179 (51.4)	170 (48.7)
Post-school qualification Complete	208 (29.8)	103 (29.6)	105 (30.1)
*Employment status*			
Not working	245 (33.5)	126 (34.0)	119 (33.1)
Working part time, casual, unknown hours	197 (27.0)	108 (29.1)	89 (24.7)
Working full time	289 (39.5)	137 (36.9)	152 (42.2)
Health			
Self-rated health (poor)	18 (2.6)	9 (2.5)	9 (2.7)
Smoker	116 (15.0)	49 (12.6)	67 (17.4)
Current asthma	77 (10.6)	37 (10.0)	40 (11.2)

Data are mean ± SD or n (%) per group

### Amounts of sedentary time and physical activity

The amounts of sedentary time and physical activity observed in young adults are shown in Table 2. Overall, mean (±SD) waking wear time was 15.0 ± 1.6 h/day, most of which was spent sedentary (9.2 ± 1.6 h/day, 61.4 %) or in light intensity activity (5.2 ± 1.5 h/day, 34.6 %) with little time spent in moderate intensity activity (33.8 ± 25.2 min/day, 3.7 %) and very limited time spent in vigorous intensity activity (2.5 ± 5.3 min/day, 0.3 %). On average young adults spent 2.1 ± 1.2 h/day sedentary for prolonged periods of ≥30 min continuously at a time.

**Table 2:** Accelerometer-measured sedentary time and physical activity in young Australian adult women and men^a^

	All (*n* = 773)	Women (*n* = 389)	Men (*n* = 384)	*p* value*
Waking wear time, h/day	15.0 ± 1.6	15.0 ± 1.5	14.9 ± 1.6	.584
Sedentary, % of waking wear	61.4 ± 10.1 %	62.8 ± 9.0 %	60.0 ± 11.0 %	<.001
Light activity, % of waking wear	34.6 ± 9.1 %	33.8 ± 8.2 %	35.3 ± 9.8 %	<.001
Moderate activity, % of waking wear	3.7 ± 2.8 %	3.1 ± 2.5 %	4.4 ± 3.0 %	<.001
Vigorous activity, % of waking wear	0.3 ± 0.6 %	0.3 ± 0.6 %	0.2 ± 0.5 %	.010
Sedentary time, h/day	9.2 ± 1.6	9.4 ± 1.5	8.9 ± 1.7	.178
Light activity, h/day	5.2 ± 1.5	5.1 ± 1.4	5.3 ± 1.6	.002
Moderate activity, min/day	33.8 ± 25.2	28.4 ± 22.3	39.1 ± 26.7	<.001
Vigorous activity, min/day	2.5 ± 5.3	2.6 ± 5.9	2.3 ± 4.7	.020
Uncensored step counts, n/day	14758 ± 508	14002 ± 4220	15522 ± 5743	<.001
Prolonged sedentary time (in ≥30 min bouts), h/day	2.1 ± 1.2	2.1 ± 1.1	1.9 ± 1.2	.345
*MVPA, min/day*				
All minutes	36.2 ± 27.5	31.1 ± 24.6	41.4 ± 29.2	<.001
In modified bouts ≥10 min^b^	14.4 ± 19.2	12.5 ± 17.5	16.4 ± 20.7	<.001
In strict bouts ≥10 min	7.9 ± 12.3	7.2 ± 11.2	8.6 ± 13.3	.036
*% averaging ≥150 min/week MVPA*				
All minutes	47.25 ± 26.88	42.10 ± 25.18	51.66 ± 27.54	<.001
In modified bouts ≥10 min^b^	20.23 ± 20.88	14.38 ± 19.59	21.65 ± 21.83	.004
In strict bouts ≥10 min	11.15 ± 13.74	10.91 ± 12.78	11.35 ± 14.53	.234
*Accelerometer counts/min, cpm*				
All waking wear time, cpm	317 (244–399)	296 (229–367)	337 (256–442)	<.001
Sedentary time, cpm	15 (13–18)	15 (13–17)	15 (12–18)	.038
Light activity, cpm	545 (502–598)	535 (489–575)	559 (515–619)	<.001
Moderate activity, cpm	2922 (2684–3234)	2914 (2651–3182)	2927 (2700–3260)	.142
Vigorous activity, cpm	6798 (6239–6798)	6972 (6310–8006)	6614 (6200–7584)	.026
MVPA, cpm	3031 (2712–3535)	3009 (2685–3557)	3059 (2741–3531)	.565

Data are mean ± SD or median (25^th^–75^th^ percentile) Abbreviations: SD standard deviation; MVPA (moderate to vigorous physical activity); cpm counts per minute

^a^ActiGraph, GT3X+ accelerometer, low-frequency extension, minute epochs classed as: sedentary (<100 cpm) light (100–1951 cpm), moderate (1952–5274 cpm) or vigorous (≥5275 cpm)

^b^modified bouts with at least 10 min ≥ MVPA threshold, allowing up to 2 min below the MVPA threshold per bout

*p for difference between men and women, significant *p* < .05, by t test (for mean ± SD) or Mann-Whitney U test (for medians)

When activity was expressed as a percentage of waking wear time, compared with young men, young women spent more time sedentary and undertook more vigorous activity, but spent less time in light activity, moderate activity and in MVPA (Table 2). Some gender differences were very small, such as 0.3 min/day for vigorous activity, whilst others were more pronounced, such as the 10.7 min/day difference in MVPA and 29.8 min/day difference in sedentary time. Consistent with the light activity and MVPA findings the average daily step counts (uncensored) were also significantly lower in women than men (*p* < 0.001), by approximately 1500 steps. Men and women did not differ significantly in prolonged sedentary time.

Using an “every minute counts” method, nearly all (99.7 %, *n* = 771) young adults performed at least some MVPA, with a mean of 36.2 ± 27.5 min/day and a median of 30.1 min/day. By this same definition, participants’ average daily MVPA was consistent with achieving the recommendation of ≥150 min per week in 67.8 % (95 % CI: 64.6, 71.1) of young adults overall (62.2 % (95 % CI: 57.9, 68.0) of young women and 73.4 % (95 % CI: 69.3, 77.1) of young men). While all participants did at least some MVPA counting every minute, 25.2 % engaged in no MVPA over the entire assessment period that was sustained for at least ten minutes at a time, even allowing for a two-minute hesitation. The percentage of participants averaging ≥10000 steps/day based on uncensored step counts was 83.3 % (*n* = 324) in women and 83.9 % (*n* = 322) in men. Uncensored step counts with our accelerometer model and settings have been estimated to be ≈ 36 % higher than actual steps [40]; allowing for this over-estimation, a lesser but still substantial proportion of women 54.2 % (*n* = 211) and men 58.9 % (*n* = 226) had average daily step counts of 13600 or higher.

The mean intensity of the time spent in bouts classed within each of the broad intensity categories varied from person to person, with wide interquartile ranges (Table 2). Women’s sedentary time and light activity time were spent at a significantly lower average intensity than men’s, while their vigorous activity was spent at a higher average intensity than men. There was no significant or large gender difference in the intensity of their moderate activity or MVPA (most of which was moderate activity).

### For how long at a time are sedentary time and physical activity accumulated?

**Fig. 1:**
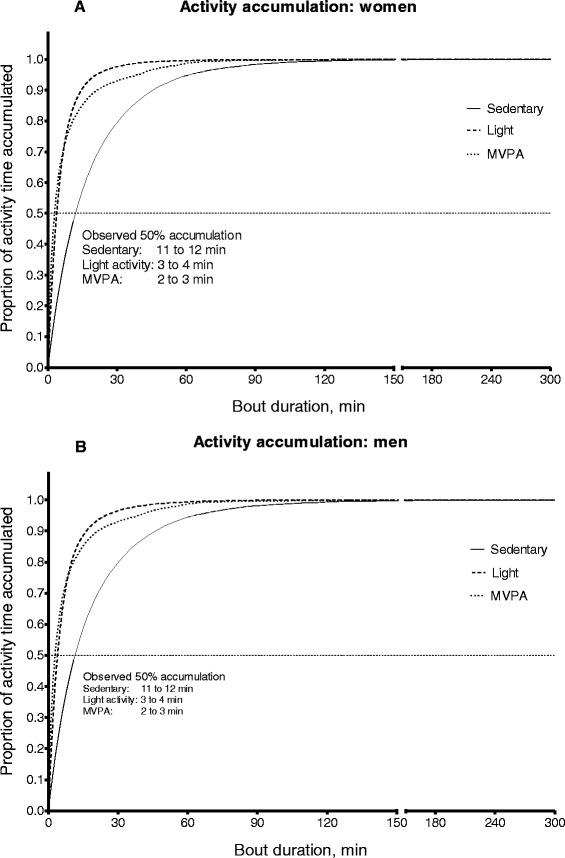
Percentage of sedentary, light and MVPA time accrued in bouts of increasing duration in all young women (*n* = 389) (**a**) and young men (*n* = 384) (**b**)

Figure 1 shows the percentage of sedentary time that young women (2a) and men (2b) were observed to accrue in bouts of increasing durations, and the theoretical accumulations of sedentary time across the entire sample of young men and young women are depicted in Additional file 1: Figure S1. On average (mean ± SD), both young men and women accumulated approximately half of their sedentary time in bouts of just under 12 min or shorter (11.8 ± 4.5 min in young adult women; 11.7 ± 5.2 min in young adult men; *p* = 0.041 for difference; Additional file 1: Figure S1). Sedentary bouts of at least 20 min and at least 30 min duration respectively accounted for 34.5 % and 21.5 % of all of the sedentary time observed in this sample of young women and 33.7 % and 21.1 % of all of the sedentary time observed in this sample of young men (Fig. 1).

Young adult men and women accumulated light activity and MVPA time in much shorter periods at a time than they accumulated their sedentary time. Half of all young men’s and young women’s light activity was accumulated in bouts of approximately 3–4 min or shorter, 25 % in bouts of approximately 1–2 min or shorter, and another 25 % accumulated in bouts longer than ≈ 8–9 min (men) or ≈ 7–8 min (women). For both young men and women, 50 % of all MVPA time that occurred was accumulated in bouts of approximately 2–3 min or shorter, with the shortest bouts (1 min) accounting for 26 % of MVPA time, and another 25 % of MVPA in bouts of approximately 8–9 min or longer. Specifically, only 22 % and 23 % of MVPA time that occurred in this sample of young men and women, respectively, was accrued in strict bouts of ≥ 10 min continuously.

**Fig. 2:**
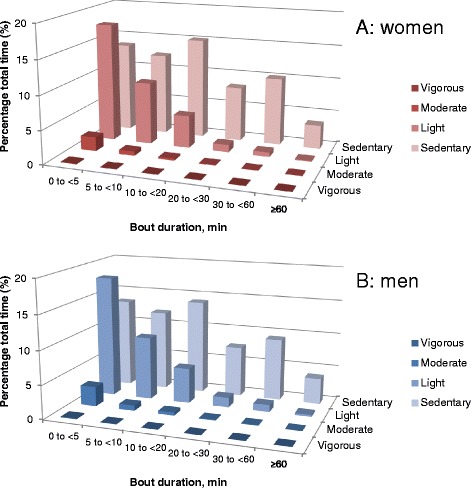
Mean daily percentage of waking wear time spent at various activity intensities (sedentary, light, moderate, vigorous) for continuous periods at a time of 0 to <5, 5 to <10, 10 to <20, 20 to <30, 30 to <60 and ≥60 min. Young women are shown in panel (**a**) and young men in panel (**b**)

Figure 2 depicts young women’s and young men’s waking wear time spent sedentary and in light, moderate and vigorous intensity accumulated for different periods at a time (EVA). Within both men and women, the dominance of sedentary time over other intensities is highlighted in the figure, as is the dominance of short bouts of light and moderate intensity activity.

### Which times of day and on which days are young adults sedentary and physically active?

**Fig. 3:**
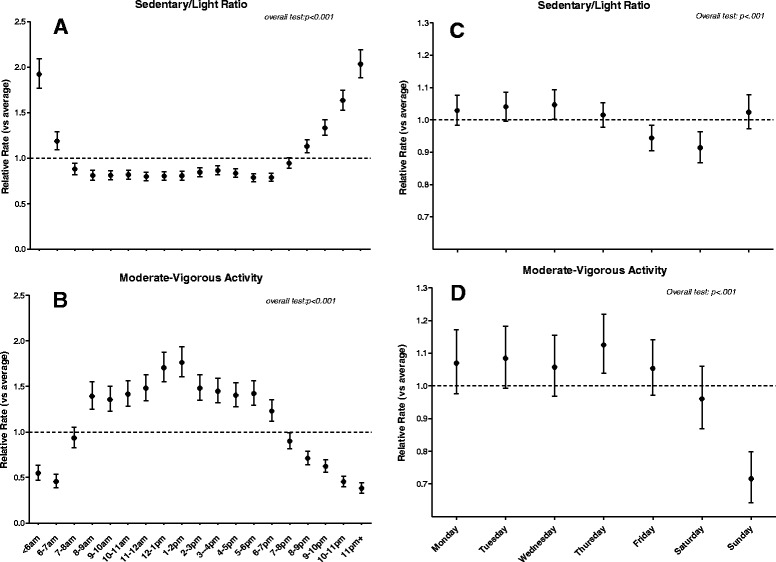
Hourly and day to day variation in sedentary/light ratio (**a**, **c**) and moderate-vigorous activity (**b**, **d**) across hours of the day and days of the week. Data are relative rates (and Sidak adjusted 95%CI) for each hour of the day or day of the week vs the grand mean. Dashed line represents the grand mean. *Note:* All models adjust for gender (male/female), ethnicity (Caucasian/other), individual income after tax (<AUD$1076/week/≥AUD$1076/week or unknown), living arrangements (alone/with others (excluding partner and parents)/with partner/with parents (and not with partner)) education current and achieved, (high school or less & not studying / high school or less & currently studying/completed TAFE, college, other or completed university/ unknown), employment status (not working/working part time, casual or unknown hours/working full-time), self-rated health, current asthma (yes/no or unknown), smoking (yes/ no or missing

There was significant and large variation by hour of the day in mean sedentary/light ratio as well as the odds of young adults performing MVPA (both *p* < 0.001), as shown in Fig. 3a and 3b respectively. The adjusted mean probability of engaging in MVPA (Fig. 3b) was above average for all hours between 08:00 and 18:59 (23 to 76 % higher in relative terms, Relative Rate (RR) = 1.23 to 1.76) and below average both from 19:00 onwards and before 07:00 (11 % lower to 2.6-fold lower, RR = 0.90 to 0.38), with only the hour 07:00–07:59 not varying significantly from the overall mean (RR = 0.94, 95 % CI: 0.83, 1.05, *p* = .819). The balance of time spent sedentary versus in light activity (as a ratio) (Fig. 3a) excludes MVPA time, but still showed an hourly patterning similar to MVPA. Young adults were less sedentary than average over the period 07:00–18:59 (hourly means were 14 to 27 % lower, RR = 0.88 to 0.79) and were more sedentary than average both from 20:00 onwards and before 07:00 (means 13 % to 2-fold higher, RR = 1.13 to 2.03). Sedentary/light ratio did not vary significantly (*p* = .092) or substantially from the overall average for the 19:00–19:59 h (RR = 0.95, 95 % CI: 0.89, 1.00).

Figures 3c and d display the difference in the geometric means for each day of the week relative to the overall mean across the week, having accounted for differences in the types of participants providing data on each day. Sedentary/light ratio (Fig. 3c) differed significantly by day of the week, by small amounts. Fridays and Saturdays were both less sedentary than average (respectively, by 6 % and 9 %, RR = 0.94, 95 % CI: 0.91, 0.98 and RR = 0.91, 95 % CI: 0.87, 0.96) while Wednesdays were more sedentary than average (by 5 %, RR = 1.05, 95 % CI: 1.00, 1.09). Other differences were small and non-significant from the overall mean (1 to 3 % higher or lower). Mean MVPA (Fig. 3d) varied significantly by day of the week (*p* = <0.001), being below average on Sundays, by approximately 30 % (RR = 0.72, 95 % CI: 0.64, 0.80), and being above average on Thursdays, by 13 % (RR = 1.13, 95 % CI: 1.04, 1.22). On the other days, the differences were not statistically significant and were small, ranging from 4 % below to 8 % above average (RR = 0.96 to 1.08).

Examining the random variation, that is how values on one day are related to values on the other days (Additional file 1: Table S2), the correlations tended be stronger for any two weekdays (*r* = 0.37 to 0.50 for MVPA and *r* = 0.42 to 0.59 for sedentary/light ratio) or for two weekend days (*r* = 0.41 for MVPA and *r* = 0.44 for sedentary/light ratio) than for weekday–weekend day pairings (*r* = 0.23 to 0.35 for MVPA and *r* = 0.24 to 0.36 for sedentary/light ratio). The sole exception was that Fridays and Saturdays displayed a moderately strong correlation (*r* = 0.46) in sedentary/light ratio.

## Discussion

This study provides the first detailed report of young women’s and young men’s activity across the spectrum, from sedentary through to light-, moderate- and vigorous- intensity, including the patterns of these activities in terms of bout durations and when over the course of a day and week these activities were undertaken. The young adults in this study were sedentary for more than half of their waking day, accumulated their sedentary time in longer bout durations than light activity or MVPA, and varied their sedentary time and physical activity by time of day and day of the week. Young women differed from young men in some but not all aspects of their activity.

The average sedentary time for the 22 year olds in the present study (9.2 h/day of 15.0 h/day waking wear time; 61 %) was slightly higher than that observed in 300 young adults aged 20–29 years in the NHANES survey (7.5 of 13.9 h/day waking wear; ≈54 %) [6] and the 8.4 of 14.6 h/day (58 %) observed in Belgian adults aged 20–65 years and slightly under the estimates reported in Canadian men and women aged 20–39 years of 9.5 h/day of 14.8 h/day (64 %) and 9.5 of 14.5 h/day (66 %), respectively [22, 23]. In 5192 Mexicans aged 18–34 years, 72 % of their time was spent sedentary (11.4 of 15.9 h/day) [24]. In addition to possible true differences between the populations of young adults (of varying ages), methodological differences could also contribute to the differences observed. Specifically, prior studies (NHANES and Canadian study) used waking wear protocols (we used continuous wear), and differed from the current study in accelerometers used (Actical in the Canadian study and Latino study), accelerometer models (Actigraph 7164 in NHANES), filter settings and/or non-wear data processing procedures.

Despite methodological and other differences, all available studies suggest that young adults spend more than half of their waking time being sedentary. Whilst there are as yet no sedentary behaviour guidelines to determine the proportion of young adults exposed to *excessive* sedentary behaviour, evidence [8] suggests sedentary behaviour may be a substantial health risk for many young people.

The average time spent in moderate intensity activity by young women (28.4 min/day) and men (39.1 min/day) in this study was broadly similar to the few other studies on young adults. Specifically, a study of 431 women and men from the US aged 20–29 years (from the NHANES survey) accumulated 22.4 min/day and 37.9 min/day of MVPA respectively. Similar estimates of time have been reported for Canadian women (24 min/day) and men (33 min/day) aged 20–39 years for a 14.1 h/day [22].

There are key differences between the tools used to generate evidence for international physical activity guidelines (self-report of specific, deliberate activities maintained for a minimum period) and accelerometer methods (movement in any context measured over a short period such as a minute). There are currently no methods to appropriately harmonise evidence collected from accelerometers with self-report based guidelines [41]. Self-report estimates have placed the prevalence of young adult Australians (18–24 year olds) who participate in at least 150 min of MVPA per week, at 57 % (Australian Bureau of Statistics 2014). Similar proportions of young adults (18–24 year olds) meeting this commonly used guideline have been reported in women (53 %) and men (57 %) from 29 European countries [42]. In our study, counting every minute above the moderate threshold as MVPA, more than two thirds (68 %) of the participants had mean daily MVPA over the monitoring period that was consistent with achieving the recommended 150 min or more of MVPA per week [43]. When considering MVPA in sustained bouts of ten or more minutes, either allowing for two minute hesitation period or examining strictly continuous periods, fewer participants achieved activity levels that were consistent with guidelines (22.8 % and 11.4 % overall, and varying somewhat between women and men). Average daily steps, after taking into account an estimated ≈ 36 % overestimation for unfiltered step counts with the accelerometer model and filter setting used [40], are consistent with the recommendation of at least 10,000 steps per day [44] for 54.2 % of young women and 58.9 % of young men. The current study shows, consistently across a range of methods, that there is a substantial proportion of young adults whose activity does not meet recommended levels and who are therefore likely at increased risk of a variety of health issues including coronary heart disease, metabolic syndrome, type 2 diabetes, breast and colon cancer, depression and falling [45]. Though little evidence has been derived from tracking the same young adults over time, the tendency seen in cross-sectional studies for time spent in MVPA to decrease with age [20, 22] suggests, in the absence of effective interventions, that the risk profile for groups of young adults like this is more likely to worsen as they move into middle age.

In the present study, women spent ≈ 2–3 % more of their waking day (about 30 min/day in total) sedentary than men. Their sedentary time was also accumulated for slightly longer periods at a time and at a lower average intensity, suggesting this difference is not entirely due to misclassified stationary light activities (in which case one might expect more sedentary time, but at a higher average intensity and/or interrupted more regularly). The finding is consistent with the US NHANES 2003–2004 findings from the young adult group (20–29 years), in which young women spent about 25 min more per day in sedentary time than young men [6], but differs from the Canadian findings of a non-significant difference of 1 min/day in the sedentary time of young women and men aged 20–39 years [22].

In the current study women compared with men had slightly higher amounts of vigorous activity and performed their vigorous activity at a higher average intensity (in terms of accelerometer counts per minute). Given the consistency in findings between amounts and average intensity, the result is unlikely due to use of a vigorous cut-point that is too low. In contrast, in the NHANES study and Canadian studies within the young adult age bracket (20–29 or 20–39 years), women had lower time spent in vigorous activity than men, although by a small amount [20, 22]. Consistent with the current study, these previous studies reported less MVPA in women than men.

Much of the young adults’ sedentary time in the current study was accumulated in the form of prolonged bouts (34.1 % in bouts ≥20 min and 21.3 % in bouts ≥30 min); findings which are similar to those from adults aged 18 and older in the NHANES study (33.7 % of accrued in bouts ≥20 min and 21.5 % in bouts ≥30 mins or more) [46]. The usual bout durations of sedentary time were slightly higher for women than men, but consistently averaged just over 11 min. These figures should not be taken to indicate that young adults only typically sit for around ten minutes at a time; interruptions to accelerometer bouts of sedentary time occur far more frequently than actual sitting to standing postural transitions [47]. In contrast to sedentary time, half of all time spent in bouts of light activity and MVPA were very short (≤3–4 min and ≤2–3 min respectively). Less than a quarter of all MVPA time was accrued in bouts of 10 or more minutes. The health-related importance of these findings relate to current evidence suggesting that sedentary time accrued in prolonged bouts increases cardio-metabolic disease risks [15, 48, 49]. Accordingly, a useful focus of health programs could be to identify novel ways to encourage young adults to shorten bouts of time spent sedentary and prolong bouts of time spent in MVPA.

The diurnal patterning of both MVPA and sedentary/light ratio showed sedentariness to reduce (and MVPA to increase) across the day and then to increase (and MVPA to reduce) again in the evening. Such diurnal patterns have been observed in diverse populations, including children [50] and older adults [51], but have not been previously described in young adults. Day of the week is sometimes associated with activity, with office workers spending 8 % more time sedentary on work days than non-work days [52], school students showing similar amounts of sedentary time on school days and weekend days [53], and a recent study of colon cancer survivors finding Saturdays were most sedentary and no significant differences in MVPA by day of the week [54]. Daily patterning in the young adults in the current study was not as simple as active weekends and sedentary weekdays, instead being more consistent with the concept that Wednesday is “hump day” (Wednesday was highly sedentary) and Thursday the unofficial beginning of the weekend [55]. Sunday through Wednesday tended to be less active and/or more sedentary while the period Thursday through Saturday tended to be more active and/or less sedentary.

The variation observed by day of the week has methodological and practical implications. A single daily average from an incomplete week has flaws as an approach to estimating usual activity for an individual, particularly for MVPA, as it ignores systematic variability. Most estimates of the number of days of monitoring required to produce habitual estimates for an individual assume day to day error is random and any two days share the same correlation as any two other days [56]; neither of these assumptions held true for the young adults in the current study. Longer periods of monitoring are likely needed to determine usual activity for an individual young adult. Practically, such day to day variation suggests that there are external factors commonly part of young adults’ weekly routines that either increase or decrease the chances of engaging in sedentary behaviours, light intensity activities and MVPA. Our findings offer interesting possibilities for targeted interventions, with time periods with the worst profile perhaps offering the most “room to improve”, for example, by replacing evening sitting with activity [57], or alternatively the time periods with better profiles may reflect times when activity is easier to undertake. Qualitative research may be useful to understand why young adults engage in sedentary behaviours and physical activity at particular times and days.

### Strengths and limitations

A key contribution of this study is the detailed reporting of young adults’ sedentary time, light activity, moderate activity and vigorous activity; including the patterns in terms of bouts and when over the course of a day and week they perform these behaviours. The pattern of accumulation of sedentary time and physical activity is emerging as important, but had yet to be described specifically in young adults. The sample was of moderate size and reasonably representative of the spread of a number of sociodemographic features, although with some proportion differences, of a group of racially homogenous young adults of around 22 years of age in a particular geo-cultural location. The use of hip-worn Actigraph accelerometer data in one minute epochs was typical of much of the literature [58], enhancing comparability of findings. However, such methods have limitations in measurement of activity, including under-detection of non-ambulatory physical activities, and limited validity for assessing patterns of sedentary accumulation [47]. Though cut-point choice was an unlikely explanation for the gender differences observed (which were sometimes counter to the literature), the broader issue of misclassified activity types in hip-worn accelerometer data could affect comparisons between men and women. For example, if well captured activities (e.g., jogging/running) comprise more of young women’s than young men’s vigorous activity, this would contribute to the higher amounts and intensity of vigorous activity observed in women than men.

## Conclusions

Young adults spent much of their day sedentary, despite many also engaging in levels of MVPA consistent with self-report based public health recommendations over their monitored period. It will be important for future studies to identify correlates and life-course predictors of sedentary behaviours and physical activity specifically in young adults to help inform targets for early interventions. Young adults are accumulating a substantial proportion of their sedentary time in prolonged periods and differently across various times of the day and days of the week. The findings on the accumulation patterns of sedentary time and physical activity can be used to inform targeted interventions in order to try and improve the current behaviours of young adults, and thus their life-course trajectories for activity related behaviours and health sequelae.

## Additional file

Additional file 1: Table S1. Socio-demographic comparison of participants in this study (*n* = 773) (Raine year 22 follow up) with contemporaneous Western Australian 22 year olds (2011 Census data). **Table S2.** Correlations matrix showing correlations between days of the week in linear mixed models examining daily variation in sedentary time (sedentary/light ratio) and MVPA. **Figure S1.** Sedentary accumulation in all Raine young women (top) and men (bottom), with usual sedentary bout duration [59]. (DOCX 110592 kb)

## References

[1] Nelson MC, Story M, Larson NI, Neumark-Sztainer D, Lytle LA (2008). Emerging adulthood and college-aged youth: an overlooked age for weight-related behavior change. Obesity (Silver Spring).

[2] Kwan MY, Cairney J, Faulkner GE, Pullenayegum EE (2012). Physical activity and other health-risk behaviors during the transition into early adulthood: a longitudinal cohort study. Am J Prev Med.

[3] Dierker L, Lloyd-Richardson E, Stolar M, Flay B, Tiffany S, Collins L, Bailey S, Nichter M, Nichter M, Clayton R, Tobacco Etiology Research N (2006). The proximal association between smoking and alcohol use among first year college students. Drug Alcohol Depend.

[4] Telama R, Yang X, Leskinen E, Kankaanpaa A, Hirvensalo M, Tammelin T, Viikari JS, Raitakari OT (2014). Tracking of physical activity from early childhood through youth into adulthood. Med Sci Sports Exerc.

[5] Hull E, Rofey D, Robertson R, Nagle E, Otto A, Aaron D (2010). Influence of marriage and parenthood on physical activity: A 2-Year Prospective Analysis. J Phys Act Health.

[6] Matthews CE, Chen KY, Freedson PS, Buchowski MS, Beech BM, Pate RR, Troiano RP (2008). Amount of time spent in sedentary behaviors in the United States, 2003–2004. Am J Epidemiol.

[7] Lee IM, Shiroma EJ, Lobelo F, Puska P, Blair SN, Katzmarzyk PT (2012). Effect of physical inactivity on major non-communicable diseases worldwide: an analysis of burden of disease and life expectancy. Lancet.

[8] Biswas A, Oh PI, Faulkner GE, Bajaj RR, Silver MA, Mitchell MS, Alter DA (2015). Sedentary time and its association with risk for disease incidence, mortality, and hospitalization in adults: a systematic review and meta-analysis. Ann Intern Med.

[9] Begg S, Vos T, Barker B, Stevenson C, Stanley L, Lopez A (2007). Burden of disease and injury in Australia, 2003.

[10] Van der Ploeg H, Chey T, Korda R, Banks E, Bauman A (2012). Sitting Time and All-Cause Mortality Risk in 222 497 Australian Adults. Arch Intern Med.

[11] Buman M, Winkler E, Kurka J, Hekler E, Baldwin C, Owen N, Ainsworth B, Healy GN, Gardiner PA (2014). Reallocating Time to Sleep, Sedentary Behaviors, or Active Behaviors: Associations With Cardiovascular Disease Risk Biomarkers, NHANES 2005–2006. Am J Epidemiol.

[12] Carson V, Ridgers ND, Howard BJ, Winkler EA, Healy GN, Owen N, Dunstan DW, Salmon J (2013). Light-intensity physical activity and cardiometabolic biomarkers in US adolescents. PLoS One.

[13] Zick CD, Stevens RB, Bryant WK (2011). Time use choices and healthy body weight: a multivariate analysis of data from the American Time Use Survey. Int J Behav Nutr Phys Act.

[14] Healy GN, Clark BK, Winkler EA, Gardiner PA, Brown WJ, Matthews CE (2011). Measurement of adults' sedentary time in population-based studies. Am J Prev Med.

[15] Dunstan DW, Kingwell BA, Larsen R, Healy GN, Cerin E, Hamilton MT, Shaw JE, Bertovic DA, Zimmet PZ, Salmon J, Owen N (2012). Breaking up prolonged sitting reduces postprandial glucose and insulin responses. Diabetes Care.

[16] De Bourdeaudhuij I, Sallis J, Vandelanotte C (2002). Tracking and explanation of physical activity in young adults over a 7-year period. Res Q Exerc Sport.

[17] Caspersen C, Pereira M, Curran K (2000). Changes in Physical Activity Patterns in the US by Sex and Cross Sectional Age. Med Sci Sports Exerc.

[18] Biddle SJ, Pearson N, Ross GM, Braithwaite R (2010). Tracking of sedentary behaviours of young people: a systematic review. Prev Med.

[19] Brown WJ, Trost SG (2003). Life transitions and changing physical activity patterns in young women. Am J Prev Med.

[20] Troiano RP, Berrigan D, Dodd KW, Masse LC, Tilert T, McDowell M (2008). Physical activity in the United States measured by accelerometer. Med Sci Sports Exerc.

[21] Colley RC, Garriguet D, Janssen I, Craig CL, Clarke J, Tremblay MS (2011). Physical activity of Canadian children and youth: accelerometer results from the 2007 to 2009 Canadian Health Measures Survey. Health Rep.

[22] Colley R, Garriguet D, Janssen I, Craig CL, Clarke J, Tremblay MS (2011). Physical activity of Canadian adults: Accelerometer results from the 2007 to 2009 Canadian Health Measures Survey. Health Rep.

[23] Van Dyck D, Cardon G, Deforche B, Owen N, Sallis JF, De Bourdeaudhuij I (2010). Neighborhood walkability and sedentary time in Belgian adults. Am J Prev Med.

[24] Merchant G, Buelna C, Castañeda SF, Arredondo EM, Marshall SJ, Strizich G, Sotres-Alvarez D, Chambers EC, McMurray RG, Evenson KR (2015). Accelerometer-measured sedentary time among Hispanic adults: Results from the Hispanic Community Health Study/Study of Latinos (HCHS/SOL). Prev Med Rep.

[25] Newnham J, Evans S, Michael C, Stanley F, Landau L (1993). Effects of frequent prenatal ultrasound on birthweight: follow up at 1 year of age. Lancet.

[26] Straker LM, Hall GL, Mountain J, Howie EK, White E, McArdle N, Eastwood PR, Raine Study 22 year follow-up Investigator G (2015). Rationale, design and methods for the 22 year follow-up of the Western Australian Pregnancy Cohort (Raine) Study. BMC Public Health.

[27] Tudor-Locke C, Barreira TV, Schuna JM, Mire EF, Katzmarzyk PT (2014). Fully automated waist-worn accelerometer algorithm for detecting children’s sleep period time separate from 24-hour physical activity or sedentary behaviors. Appl Physiol Nutr Metab.

[28] Tracy DJ, Xu Z, Choi L, Acra S, Chen KY, Buchowski MS (2014). Separating bedtime rest from activity using waist or wrist-worn accelerometers in youth. PLoS One.

[29] Kinder JR, Lee KA, Thompson H, Hicks K, Topp K, Madsen KA (2012). Validation of a hip-worn accelerometer in measuring sleep time in children. J Pediatr Nurs.

[30] Barreira TV, Schuna JM, Mire EF, Katzmarzyk PT, Chaput JP, Leduc G, Tudor-Locke C (2015). Identifying children's nocturnal sleep using 24-h waist accelerometry. Med Sci Sports Exerc.

[31] Tudor-Locke C, Barreira TV, Schuna JM, Mire EF, Katzmarzyk PT (2014). Fully automated waist-worn accelerometer algorithm for detecting children's sleep-period time separate from 24-h physical activity or sedentary behaviors. Appl Physiol Nutr Metab.

[32] McVeigh J, Winkler E, Healy GN, Slater J, Eastwood P, Straker LM: Isolating out-of-bed wear from non-wear and in-bed wear periods in young adults hip-worn accelerometer data (continuous wear protocol). In International Conference on Diet and Activity Methods. Brisbane, Australia; 2015

[33] McVeigh J, Winkler E, Healy GN, Slater J, Eastwood P, Straker L: Validity in young adults of automated detection of waking wear from hip-worn accelerometer data with a continuous wear protocol. In International Conference on Ambulatory Monitoring of Physical Activity and Movement. Limerick, Ireland; 2015

[34] Rich C, Geraci M, Griffiths L, Sera F, Dezateux C, Cortina-Borja M (2013). Quality control methods in accelerometer data processing: defining minimum wear time. PLoS One.

[35] Freedson PS, Melanson E, Sirard J (1998). Calibration of the Computer Science Applications, Inc. accelerometer. Med Sci Sports Exerc.

[36] Glazer NL, Lyass A, Esliger DW, Blease SJ, Freedson PS, Massaro JM, Murabito JM, Vasan RS (2013). Sustained and shorter bouts of physical activity are related to cardiovascular health. Med Sci Sports Exerc.

[37] Hagstromer M, Troiano RP, Sjostrom M, Berrigan D (2010). Levels and patterns of objectively assessed physical activity--a comparison between Sweden and the United States. Am J Epidemiol.

[38] Straker L, Campbell A, Mathiassen SE, Abbott RA, Parry S, Davey P (2014). Capturing the pattern of physical activity and sedentary behavior: exposure variation analysis of accelerometer data. J Phys Act Health.

[39] Chapman N, Hill K, Taylor S, Hassanali M, Straker L, Hamdorf J (2014). Patterns of physical activity and sedentary behavior after bariatric surgery: an observational study. Surg Obes Relat Dis.

[40] Feito Y, Garner HR, Bassett DR (2015). Evaluation of ActiGraph's low-frequency filter in laboratory and free-living environments. Med Sci Sports Exerc.

[41] Loney T, Standage M, Thompson D, Sebire S, Cumming S (2011). Self-report vs. objectively assessed physical activity: Which is right for public health?. J Phys Act Health.

[42] Marques A, Sarmento H, Martins J, Saboga Nunes L (2015). Prevalence of physical activity in European adults - Compliance with the World Health Organization's physical activity guidelines. Prev Med.

[43] Brown WJ, Bauman AE, Bull FC, NWB. Development of Evidence-based Physical Activity Recommendations for Adults (18-64 years). Evidence-based Physical Activity Recommendations for Adults (18-64 years). Report prepared for the Australian Government Department of Health, August 2012; 2012

[44] Tudor-Locke C, Bassett DR (2004). How many steps/day are enough? Preliminary pedometer indices for public health. Sports Med.

[45] Zhao G, Li C, Ford ES, Fulton JE, Carlson SA, Okoro CA, Wen XJ, Balluz LS (2014). Leisure-time aerobic physical activity, muscle-strengthening activity and mortality risks among US adults: the NHANES linked mortality study. Br J Sports Med.

[46] Kim Y, Welk GJ, Braun SI, Kang M (2015). Extracting objective estimates of sedentary behavior from accelerometer data: measurement considerations for surveillance and research applications. PLoS One.

[47] Lyden K, Kozey Keadle SL, Staudenmayer JW, Freedson PS (2012). Validity of two wearable monitors to estimate breaks from sedentary time. Med Sci Sports Exerc.

[48] Healy GN, Dunstan DW, Salmon J, Cerin E, Shaw JE, Zimmet PZ, Owen N (2008). Breaks in Sedentary Time. Diabetes Care.

[49] Healy GN, Matthews CE, Dunstan DW, Winkler EA, Owen N (2011). Sedentary time and cardio-metabolic biomarkers in US adults: NHANES 2003-06. Eur Heart J.

[50] Pereira S, Gomes TN, Borges A, Santos D, Souza M, Dos Santos FK, Chaves RN, Katzmarzyk PT, Maia JA (2015). Variability and stability in daily moderate-to-vigorous physical activity among 10 year old children. Int J Environ Res Public Health.

[51] Davis MG, Fox KR (2007). Physical activity patterns assessed by accelerometry in older people. Eur J Appl Physiol Occup Physiol.

[52] Clemes SA, O'Connell SE, Edwardson CL (2014). Office workers' objectively measured sedentary behavior and physical activity during and outside working hours. J Occup Environ Med.

[53] Abbott RA, Straker LM, Mathiassen SE (2013). Patterning of children's sedentary time at and away from school. Obesity (Silver Spring).

[54] Lynch BM, Boyle T, Winkler E, Occleston J, Courneya KS, Vallance JK. Patterns and correlates of accelerometer-assessed physical activity and sedentary time among colon cancer survivors. Cancer Causes Control. 2016;27:59-68. 10.1007/s10552-015-0683-4 26518196

[55] Hump day. In The Oxford English Dictionary, 11 edition. Oxford: Oxford University Press; 2008.

[56] Baranowski T, Masse LC, Ragan B, Welk G (2008). How many days was that? We're still not sure, but we're asking the question better!. Med Sci Sports Exerc.

[57] Smith L, Hamer M, Ucci M, Marmot A, Gardner B, Sawyer A, Wardle J, Fisher A (2015). Weekday and weekend patterns of objectively measured sitting, standing, and stepping in a sample of office-based workers: the active buildings study. BMC Public Health.

[58] Wijndaele K, Westgate K, Stephens SK, Blair SN, Bull FC, Chastin SF, Dunstan DW, Ekelund U, Esliger DW, Freedson PS (2015). Utilization and harmonization of adult accelerometry data: Review and expert consensus. Med Sci Sports Exerc.

[59] Stephens SK, Winkler EA, Trost SG, Dunstan DW, Eakin EG, Chastin SF, Healy GN: Intervening to reduce workplace sitting time: how and when do changes to sitting time occur? Br J Sports Med 2014, 48:1037-1042. 10.1136/bjsports-2014-093524 24815544

