# Supplementary material for: Objectively measured patterns of sedentary time and physical activity in young adults of the Raine study cohort
Source: Int J Behav Nutr Phys Act. 2016 Mar 24;13:41. doi: 10.1186/s12966-016-0363-0 (PMC4806520; doi:10.1186/s12966-016-0363-0)
Supplement: Additional file 1: Table S1. — Socio-demographic comparison of participants in this study (n = 773) (Raine year 22 follow up) with contemporaneous Western Australian 22 year olds (2011 Census data). Table S2. Correlations matrix showing correlations between days of the week in linear mixed models examining daily variation in sedentary time (sedentary/light ratio) and MVPA. Figure S1. Sedentary accumulation in all Raine young women (top) and men (bottom), with usual sedentary bout duration [59]. (DOCX 110592 kb) [file 12966_2016_363_MOESM1_ESM.docx]

**Supplementary Table 1**. Socio-demographic comparison of participants in this study (n=773) (Raine year 22 follow up) with contemporaneous Western Australian 22 year olds (2011 Census data ).

| Variable | Women | | Men | |
| --- | --- | --- | --- | --- |
|  | Census | Raine | Census | Raine |
| Education |  |  |  |  |
| *Highest school year completed* |  |  |  |  |
| <=9 | 1.9 | 1.1 | 2.8 | 0.9 |
| 10 | 12.7 | 7.7 | 16.2 | 11.7 |
| 11 | 9.8 | 6.9 | 13.5 | 5.7 |
| 12 | 75.6 | 84.3 | 67.5 | 81.7 |
| *Current study status* |  |  |  |  |
| Not studying | 66.6 | 51.4 | 70.1 | 51.1 |
| Studying | 33.4 | 48.6 | 29.9 | 48.9 |
| Occupation and employment |  |  |  |  |
| *Labour force status* |  |  |  |  |
| Not in labour force | 22.4 | 9.8 | 15.9 | 14.6 |
| Unemployed | 5.7 | 8.0 | 7.0 | 9.4 |
| Employed | 71.9 | 82.2 | 77.1 | 76.0 |
| *Work hours per week (workers)* |  |  |  |  |
| 1-24 | 21.0 | 35.5 | 28.4 | 39.6 |
| 25-39 | 24.9 | 36.1 | 37.4 | 26.8 |
| 40 | 20.9 | 12.1 | 18.2 | 14.8 |
| >40 | 33.2 | 16.3 | 16.0 | 18.8 |
| *Income* |  |  |  |  |
| Low (<AUS $116/week) | 51.1 | 7.0 | 37.4 | 8.9 |
| Medium (AUS $ 116-604/week) | 33.9 | 51.9 | 31.4 | 39.9 |
| High (>AUS $605/week) | 15.0 | 43.2 | 31.2 | 51.5 |
| *Occupation* |  |  |  |  |
| Clerical | 22.5 | 26.1 | 4.1 | 8.8 |
| Community and  Service Workers | 26.2 | 21.1 | 9.7 | 14.2 |
| Labourers | 6.9 | 6.0 | 19.9 | 15.9 |
| Machiners | 1.2 | 0.9 | 9.1 | 6.4 |
| Managers | 3.7 | 2.5 | 3.3 | 2.7 |
| Professionals | 5.6 | 15.1 | 4.0 | 12.9 |
| Sales workers | 26.9 | 22.6 | 12.9 | 14.2 |
| Technicians and Trades Workers | 6.9 | 5.7 | 37.1 | 24.7 |
| Family |  |  |  |  |
| *Marital status* |  |  |  |  |
| Not married | 70.9 | 76.1 | 84.4 | 81.3 |
| de Facto | 21.7 | 22.8 | 13.0 | 17.8 |
| Married | 7.4 | 1.1 | 2.6 | 0.9 |
| *Number of children* |  |  |  |  |
| 0 | 85.0 | 96.4 |  |  |
| 1+ | 15.0 | 3.6 |  |  |

Data are % per group

Note: Income refers to individual income after tax

**Supplementary table 2.** Correlations matrix showing correlations between days of the week in linear mixed models examining daily variation in sedentary time (sedentary/light ratio) and MVPA.

|  | Su | M | T | W | Th | F | Sat |
| --- | --- | --- | --- | --- | --- | --- | --- |
|  | Estimated R Correlation Matrix | | | | | | |
| Sedentary/light ratio (transformed by natural log) | | | | | | | |
| Sunday (Su) | 1 |  |  |  |  |  |  |
| Monday (M) | 0.3551 | 1 |  |  |  |  |  |
| Tuesday (T) | 0.3592 | 0.5485 | 1 |  |  |  |  |
| Wednesday (W) | 0.2453 | 0.5341 | 0.5867 | 1 |  |  |  |
| Thursday (Th) | 0.2938 | 0.5147 | 0.5627 | 0.5442 | 1 |  |  |
| Friday (F) | 0.3527 | 0.4631 | 0.4157 | 0.5035 | 0.5283 | 1 |  |
| Saturday (Sat) | 0.4352 | 0.2866 | 0.2434 | 0.2492 | 0.3263 | 0.4588 | 1 |
| MVPA (transformed by natural log, having added 1 to all values ^a^) | | | | | | | |
| Sunday (Su) | 1 |  |  |  |  |  |  |
| Monday (M) | 0.2999 | 1 |  |  |  |  |  |
| Tuesday (T) | 0.2580 | 0.4224 | 1 |  |  |  |  |
| Wednesday (W) | 0.2699 | 0.4003 | 0.4957 | 1 |  |  |  |
| Thursday (Th) | 0.3544 | 0.4559 | 0.4404 | 0.4234 | 1 |  |  |
| Friday (F) | 0.3198 | 0.4289 | 0.3749 | 0.4695 | 0.4023 | 1 |  |
| Saturday (Sat) | 0.4130 | 0.3488 | 0.2432 | 0.2633 | 0.2342 | 0.3140 | 1 |

^a^ As many days involved no MVPA, a constant (=1) was added to all values to facilitate log transformation.

*Note:* Table presents variance and correlations from unstructured correlation matrix from linear mixed models testing day of the week effects, adjusting for participant sex and confounding sociodemographic characteristics

*Note:* All models adjust for sex (male or female), ethnicity (Caucasian or other), individual income after tax (<AUD$1076/wk or ≥AUD$1076/wk or unknown), living arrangements (alone or with others (excluding partner and parents) or with partner or with parents (and not with partner)) education and study, (high school or less & not studying or high school or less & currently studying or completed TAFE, college, other or completed university or unknown), work status (not working, working part time, casual or unknown hours or working full-time), self-rated health, current asthma (yes or no or unknown), smoking (yes or no or missing)

**Supplementary Figure 1:** Sedentary accumulation in all Raine young women (top) and men (bottom), with usual sedentary bout duration

*Note:* predicted curve and confidence limits from non-linear regression applying the Chastin method as described in Stephens et al [59].
